# Exploration on flexible wearable sensor motion monitoring based on novel functional polymer conjugated materials

**DOI:** 10.3389/fchem.2023.1265211

**Published:** 2023-12-01

**Authors:** Jie Zhang, Huanxiang Ding

**Affiliations:** College of Physical Education and Health, Linyi University, Linyi, Shandong, China

**Keywords:** flexible wearable sensors, new functional polymer conjugated materials, motion monitoring, sensor preparation, conjugated material synthesis

## Abstract

With the continuous development of flexible electronics, multi-functional device integration, artificial intelligence technology, etc., flexible wearable sensors are playing an increasingly important role in people’s daily motion monitoring. However, current flexible wearable sensors have problems such as low accuracy, poor real-time performance, and poor stability in motion monitoring, which seriously hinder the better application of flexible wearable sensors and are not conducive to the collection and monitoring of motion signals. To this end, this paper designed a flexible wearable sensor motion monitoring system and tested its performance through the preparation and performance research of new functional polymer conjugated materials. The research results show that the motion monitoring system designed based on the new functional polymer conjugated material flexible wearable sensor has good monitoring accuracy and real-time performance. When the output data is 500 pieces, the running monitoring accuracy reaches 95.4%, and the monitoring feedback time is 0.321 s; the high jump movement monitoring accuracy rate reaches 97%, and the monitoring feedback time is 0.287 s; the long jump movement monitoring accuracy rate reaches 96%, and the monitoring feedback time is 0.296 s. This shows that the flexible wearable sensor motion monitoring system of this paper has better performance and can meet the current demand for accuracy and real-time motion monitoring. This study highlights the impact of new functional polymer conjugated materials on flexible wearable sensors, helping to further solve the deficiencies of flexible wearable sensors in sports monitoring and promote their better development.

## 1 Introduction

As current computer technology develop rapidly, the application of flexible wearable sensors is becoming increasingly widespread (Vu Chi et al., 2021). In terms of sports and health, flexible wearable sensors can be used in golf training, shooting posture training, table tennis posture training, Taekwondo posture training, rowing monitoring, swimming technology statistics, etc (Li et al., 2018). In rehabilitation medicine, flexible wearable sensors can be used for gait analysis, lower limb rehabilitation training and more (Yao et al., 2018). Its core technology used is flexible sensing technology, which is a new type of sensing technology centered on flexible materials. It has the characteristics of light weight, comfortable wearing and high accuracy. Its application in wearable medical devices can greatly ameliorate the performance of existing sensors and be extensively applied in the medical field. However, most of the commonly used flexible wearable sensors currently use hard materials with poor flexibility, which have defects such as poor comfort and detection accuracy affected by movement speed. However, flexible wearable sensors with high detection accuracy are difficult to satisfy the demands of daily wearable and real-time sports health monitoring due to their large size, limited application and high cost (Fu et al., 2021). Therefore, this article prepared conjugated material compounds that can be applied between conductive fiber membranes and copper strips of sensors. TPU (Thermoplastic polyurethanes) fiber membrane composed of a three-dimensional network structure is formed internally through electrospinning, and conjugated materials with excellent electrochemical performance are used as conductive fillers to wrap them on the surface of TPU fibers, Ultimately, a complete three-dimensional conduction network is formed. This article intends to ameliorate the performance of flexible wearable sensors, increase the accuracy of motion monitoring, achieve real-time motion monitoring and better satisfy people’s requirements through the application of new functional polymer conjugated materials. Meanwhile, it can provide more possibilities for the preparation of sensors and offer more theoretical basis to the wider application of new functional polymer conjugated materials.

Thanks to the continuous development of current science and technology, flexible strain sensors have potential application value in electronic skin, medical monitoring, human-computer interface and other fields. The key challenge of applying strain sensors to human motion monitoring is to achieve wide strain range and full range high sensitivity simultaneously (Chu et al., 2021). Wu Qi showed a low-cost flexible pressure sensor with positive resistance pressure response based on laser engraved graphene and said that graphene positive pressure sensor had excellent capabilities and could be well applied in intelligent perception, interactive devices, real-time health, motion monitoring and other fields (Wu et al., 2020). Zhou Yujie has prepared a tensile strain sensor based on the designed crack structure and said that the sensor could be assembled to detect various human motion and physical vibration signals, showing its potential applications in intelligent devices, electronic skin and wearable medical monitors (Zhou et al., 2019). Liu Zhanxu synthesized biodegradable polyurethane and processed it with carbon nanotubes through wet spinning to form polyurethane/carbon nanotube composite fibers for strain sensors. The biodegradability of polyurethane/carbon nanotube strain sensors in phosphate buffered saline solutions was verified, providing insights for the development of biodegradable wearable electronic devices (Liu et al., 2022). Gao Wei-Chen designed and prepared a high-performance strain sensor composed of polyurethane, reduced graphene oxide, polydopamine and other materials and said that it had good super hydrophobicity, could effectively detect subtle and large human movements and had great potential in the field of flexible and wearable electronics (Gao et al., 2021). Scholars’ research on sensors and motion monitoring can enrich their theoretical content and offer more possibilities to motion monitoring, but there are also some shortcomings. Due to scholars’ focus on the practical application value of sensors in the field of motion monitoring, there has been no in-depth research on the specific points of wearable sensors applied to motion monitoring. This leads to research results being more theoretical and without practical support, and the research results cannot be well applied.

However, some scholars have different ideas. They have studied the photoelectrochemical properties and application fields of conjugated materials and believed that conjugated materials have certain application value in sensor design. MacFarlane Liam R considered that the development of π-conjugated polymers supplied an entry point for various new functional organic materials. He introduced methods for synthesizing π-conjugated polymer nanoparticles and explained the applications of the resulting nanoparticles in electronics and optoelectronics, biomedical imaging and treatment, photocatalysis and sensing (MacFarlane et al., 2021). Koenig, Josh DB have studied the photoelectric properties of four new functional π-conjugated materials. He reckoned that the selection of aryl π-conjugated nucleus had a great influence on the energy level of the highest occupied molecular orbital of the molecule, while the selection of end caps had a more profound effect on the energy level of the lowest unoccupied molecular orbital. Therefore, functional π-conjugated materials could be used in the design of electronic devices such as sensors (Koenig et al., 2022). These scholars’ research on functional conjugated materials can broaden the design ideas of wearable sensors and supply more methods for motion monitoring. However, due to its limited application value in the surface layer, there has been no in-depth exploration of flexible wearable sensors for new functional polymer conjugated materials, which makes the research less valuable as a reference.

**FIGURE 1:**
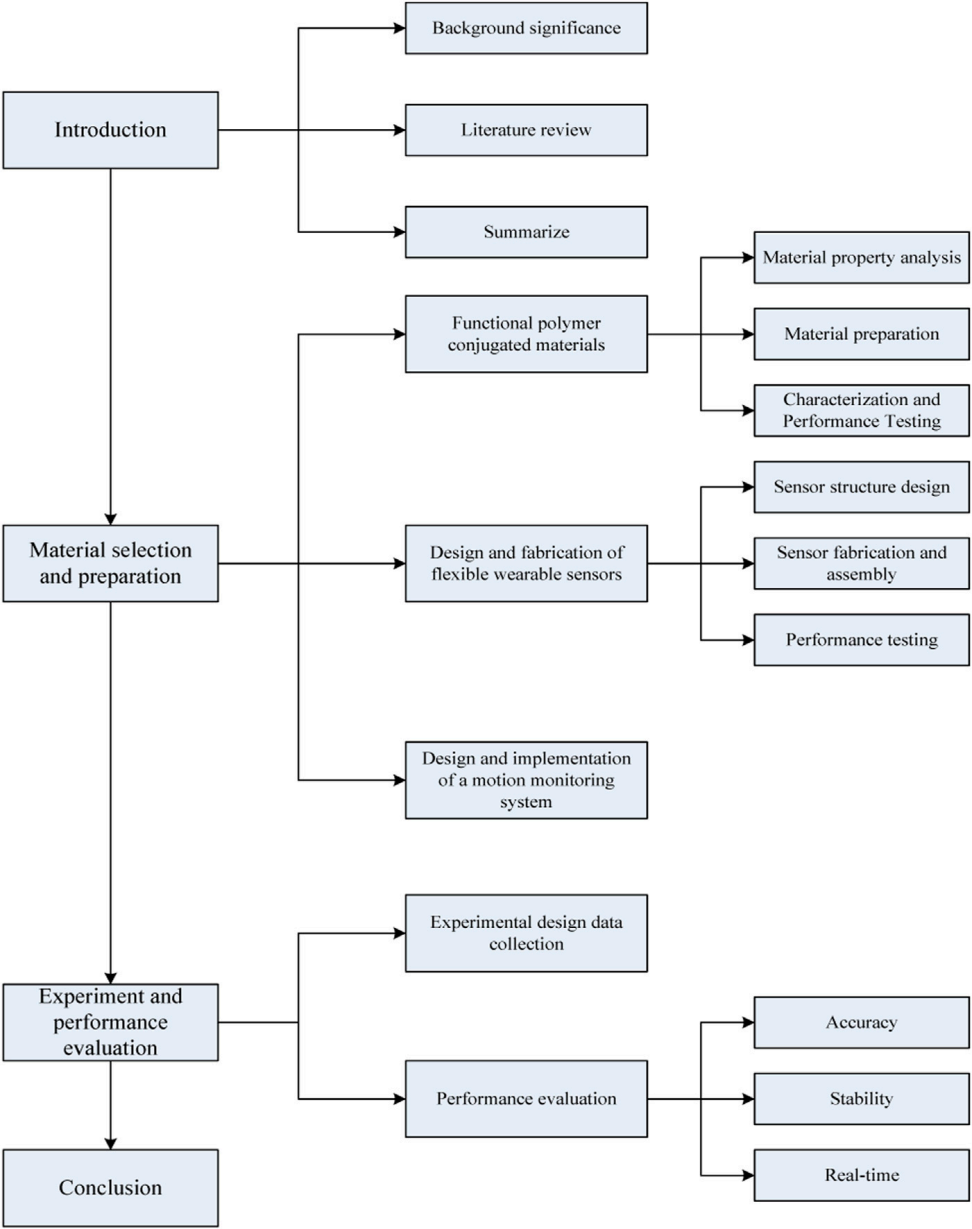
The main framework structure diagram studied in this article.

To better ameliorate the performance of flexible wearable sensors and enable them to perform better motion monitoring, this article analyzes the selection and preparation of new functional polymer conjugated materials and combines previous scholars’ discussions on sensor technology and motion monitoring to optimize the design of flexible wearable sensors. Through empirical research, it is found that the wearable sensor designed in this article has higher accuracy, better stability and real-time performance for motion monitoring. Compared with traditional sensors, the innovation of sensor design in this article lies in paying attention to the unique properties of new functional polymer conjugated materials and applying them to optimize the performance of sensors. This helps to diminish the shortcomings of sensors and ultimately better promote motion monitoring. The main framework structure of this study is shown in Figure 1.

## 2 Selection and preparation of novel functional polymer conjugated materials

### 2.1 Functional polymer conjugated materials

#### 2.1.1 Characteristic dissection

Functional polymer conjugated materials are compounds with repetitive structures composed of alternating double single bonds on the main chain of polymers. This arrangement makes the bonding and anti bonding molecular orbital nonlocalized along the main chain of the molecule (Qu et al., 2019). When π or π* bonding relies on the formation of charge transfer complexes to fill or empty, it has a higher conductivity, which is different from inorganic semiconductors. The atoms of inorganic semiconductors are bound in a three-dimensional space by covalent bond, while functional polymer conjugated materials are individual semiconductor molecules bound together in the form of a single molecule. In the same molecule, the orbits of carbon atoms overlap each other to form a molecular orbital with a π-conjugated structure and the π electrons are in the conjugated main chain delocalization. The interaction between Van der Waals force and π-π is the main form of intermolecular interaction, which plays a vital role in solid systems. The band gap of the material depends on the energy difference between the maximum occupied molecular orbital and the lowest unoccupied molecular orbital.

#### 2.1.2 Material preparation

This article refers to the preparation method of dense fluorene conjugated polymer materials, and uses chemical materials such as 1-bromo-2-ethylhexane and anhydrous potassium carbonate to prepare functional polymer conjugated materials (Xia, 2014). Due to the exploration of new chemical reaction pathways and synthesis methods for synthesizing different compounds, the development of new polymer conjugated materials can further improve the performance of sensors. Different synthesis methods can control the structure and properties of polymers, such as polymerization reaction conditions, monomer selection, catalyst type, etc. These factors can all affect the structure and properties of polymers. Therefore, synthesizing different compounds can explore new polymer conjugated materials and sensor preparation methods, improve the performance and applicability of sensors, Provide a wider range of choices and more accurate measurement methods for the field of motion monitoring. For this reason, this article divides the synthesis of new functional polymer conjugated materials into three steps, using materials such as 1-bromo-2-ethylhexane, 3-boric acid thiophene, chloroform, etc. to ultimately synthesize the target monomer compound to ensure the solubility of the target product.

First, 1-Bromo-2-ethylhexane is added to a 250 mL single neck bottle and then 100 mL of N,N-Dimethylformamide is added. Under the ice bath condition, N-bromosuccinimide is added in batches and it is determined by thin-layer chromatography. After all raw materials are converted into a single bromine product, the reaction is stopped, extracted with ether and washed with a lot of water. The organic phase could be dried through Anhydrous magnesium sulfate. The concentrated crude product is chromatographed on a silica gel column and Petroleum ether is used as eluent to obtain Compound 1 with a yield of 90%. Then, Compounds 1, 3-Thiophenylboronic acid, Tetra (triphenylphosphine) palladium, 80 mL Toluene and 40 mL Sodium carbonate are successively added to a 250 mL double neck flask. They are stirred, heated and refluxed and they all react under the action of nitrogen. The reaction is monitored by thin-layer chromatography until the reaction stops overnight and then the crude product is obtained by water washing, Petroleum ether extraction, separation, organic phase combination, drying, reduced pressure concentration. Then chromatography is carried out on the silica gel column, with Petroleum ether/ethyl acetate as eluent and finally Compound 2 is obtained, with the yield of 82%. Eventually, Compound 2 is added to a 250 mL single neck bottle and then 100 mL chloroform is added. Under the condition of ice bath, N-bromosuccinimide is added in batches and determined by thin-layer chromatography. After all raw materials are converted into a single bromine product, the reaction is stopped, extracted with chloroform. A large amount of water is used to wash and the organic phase is dried with Anhydrous magnesium sulfate. After concentration, it is chromatographed on a silica gel column. Finally, Compound 3 is obtained with Petroleum ether as eluent in 89% yield. The main chemical materials required for the experiment are shown in Table 1.

**TABLE 1:** Main chemical materials required for the experiment.

Serial number	Material name	Chemical formula	Purity level
1	Catechol	$C_{6}H_{6}O_{2}$	Chemically pure
2	Potassium carbonate	$K_{2}{\text{CO}}_{3}$	Analytical purity
3	Sodium carbonate	${\text{Na}}_{2}{\text{CO}}_{3}$	Analytical purity
4	Ethyl acetate	$C_{4}H_{8}O_{2}$	Analytical purity
5	Acetic acid	$C_{2}H_{4}O_{2}$	Analytical purity
6	Toluene	$C_{7}H_{8}$	Analytical purity
7	Trimethyltin chloride	$\text{SnCl}{\text{Me}}_{3}$	99%
8	Magnesium sulfate anhydrous	$\text{MgS}O_{2}$	Analytical purity
9	Methanol	$CH_{4}O$	Analytical purity
10	Benzophenone	$C_{13}H_{10}O$	Chemically pure
11	Sodium hydroxide	NaOH	Analytical purity
12	Chloroform	$\text{CH}{\text{CI}}_{3}$	99.7%
13	1-Bromo-2-ethylhexane	$C_{8}H_{17}\text{Br}$	95%
14	3-Thiophenylboronic acid	$C_{4}H_{5}{\text{BO}}_{2}S$	Analytical purity
15	Metallic sodium	Na	Analytical purity
16	Deuterated chloroform	$\text{CD}{\text{CI}}_{3}$	99%
17	Trio tolyl phosphate	${P\left(o-\text{tol}\right)}_{3}$	95%
18	Dichloromethane	${\text{CH}}_{2}{\text{CI}}_{2}$	Chemically pure
19	Anhydrous iron (III) chloride	$\text{Fe}{\text{CI}}_{3}$	Chemically pure
20	Tetra (triphenylphosphine) palladium	${\text{Pd}\left(\text{PP}h_{3}\right)}_{4}$	99%
21	Tri (dibenzylideneacetone) dipalladium	${Pd_{2}\left(\text{dba}\right)}_{3}$	99%

The instruments and models involved in the preparation of materials are shown in Table 2.

**TABLE 2:** Instruments and models for preparing materials.

Serial number	Instrument name	Instrument model
1	Electronic balance	CN-V3
2	Magnetic stirrer	S10-2
3	Vacuum pump	GV80
4	Rotary evaporator	RE-2000A
5	Nuclear magnetic resonance instrument	JZ-DH2002A
6	Infrared spectrometer	TENSOR27
7	Ultraviolet visible absorption spectrometer	UV-1700
8	Electrochemical analyzer	BZL-1
9	Low temperature coolant circulation pump	DLSB-80/20∼120
10	Circulating water multi-purpose vacuum pump	SHZ-95B
11	Low temperature constant temperature reaction bath	DFY-80/40
12	X-ray photoelectron spectroscopy analysis	AXIS Nova
13	Thermogravimetric analyzer	TGA-1250
14	Scanning electron microscope	SCAN-OSTEO
15	X-ray diffraction tester	Thick800A

#### 2.1.3 Material characterization and performance testing

**FIGURE 2:**
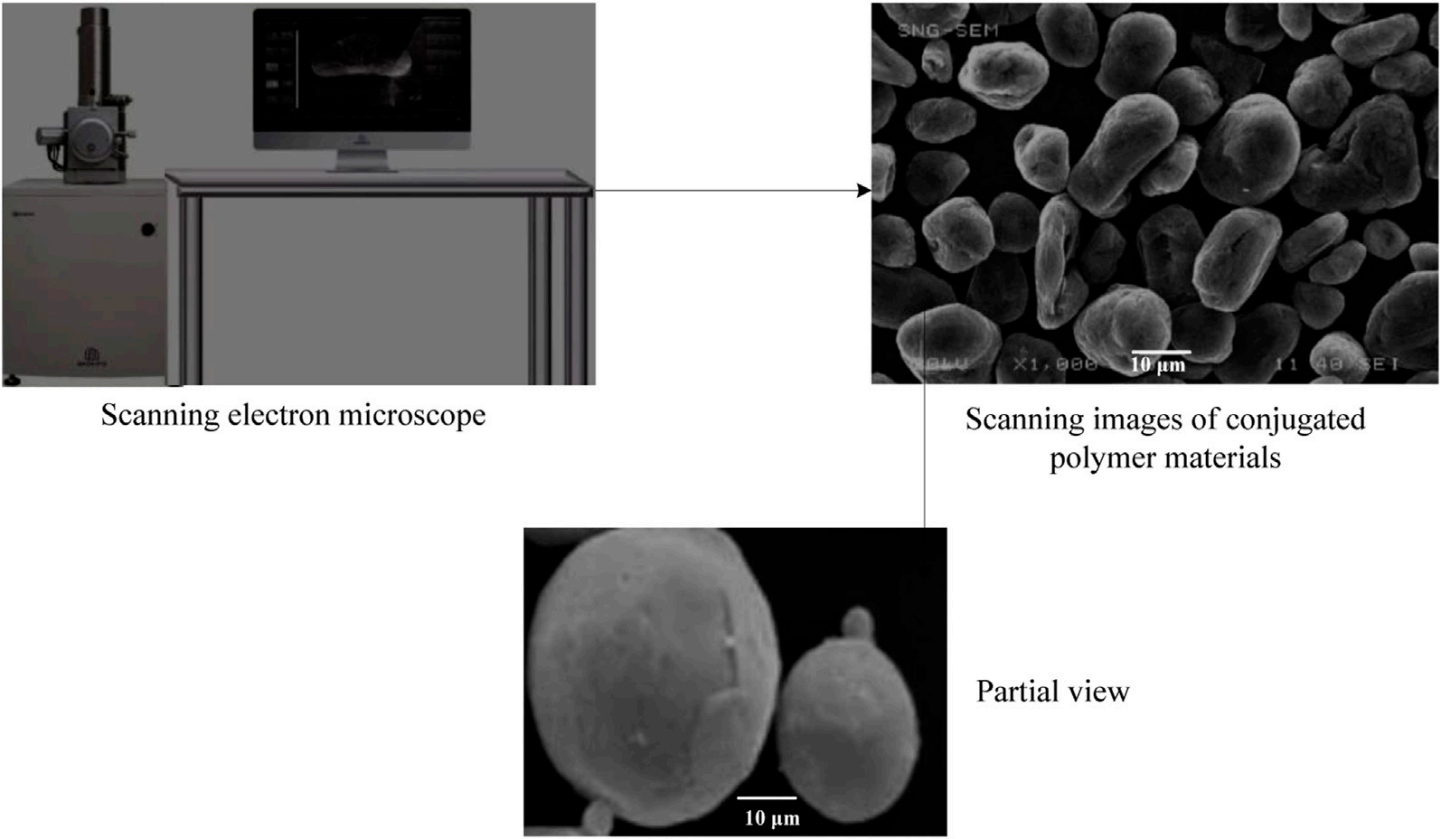
Scanning electron microscope image.

After the synthesis of three compounds, the compound performance should be tested by scanning electron microscope, thermogravimetric analyzer and diffractometer. The scanning electron microscope image is shown in Figure 2.

First, a small piece of film sample is pasted on the conductive tape and then it is pasted on the sample table. The sample is vacuumed and sprayed with platinum and then the shape of the sample is observed with scanning electron microscope. Meanwhile, the surface composition of the sample is analyzed and the X-ray diffraction pattern is obtained. Then thermogravimetric analysis is carried out and 10 mg of compound powder is selected and tested under nitrogen environment. It is necessary to adjust the temperature range to 25–800°C and heat at a rate of 10°C per minute. X-ray diffraction (XRD) method: Finally, place the sample on the sample table and perform diffraction analysis using an X-ray diffractometer. According to specific experimental requirements, set the scanning step to 0.02° and the scanning range to 2–80°. In X-ray diffraction analysis, the focus is usually on the smaller angle range, as X-ray diffraction typically occurs at smaller angles. The setting of step size will affect the accuracy and resolution of the experiment. Meanwhile, due to the fact that the detector of the selected X-ray diffractometer may not be able to receive scattered X-rays with an angle greater than 80°, its scanning range is set at 2–80°. And the setting of step size is also limited to some extent by the accuracy of the instrument. If the step size is too large, it may cause data distortion or loss. Therefore, based on specific experimental requirements and instrument limitations, we have set such a scanning range and step size.

Additionally, this article also tests the optical properties of the compound and the results are shown in Table 3.

**TABLE 3:** Optical properties of compounds.

Chemical compound		Compound 1	Compound 2	Compound 3
Solution (nanometre)	$\lambda_{\max}$	724185	679248	621325
	$\lambda_{onset}$	825	738	714
Material (film)	$\lambda_{\max}$	802192	715228	698526
	$\lambda_{onset}$	905	796	772
$\lambda_{onset}$ (nanometre)		29	16	43
$E_{g}^{opt}$		2.56	3.05	2.97

Known from Table 3, the three compounds in organic solid films are likely to produce π-π stacking during film formation owing to the presence of large π conjugated surfaces between molecules, resulting in a red shift of their absorption peaks and flipping of their shoulder peaks. Both of these results are expected to broaden the absorption spectrum of the material while reducing the bandgap width. They also indicate strong intermolecular interactions, which are beneficial for charge transfer and carrier transport.

### 2.2 Design and fabrication of flexible wearable sensors

#### 2.2.1 Sensor structure design

Flexible wearable sensors have broad application prospects in human motion monitoring, health restoration, human-computer interaction and other fields (Xie et al., 2019; Zhao et al., 2022). It can track and monitor the movement of human joints and muscles and can respond to pulse beating, throat vocalization, micro expression and others (Liu et al., 2022). It has shown great potential in intelligent kits for children, athletes and soldiers, as well as rehabilitation monitoring for chronic diseases.

The composition of a sensor usually consists of three parts, namely: sensitive components, conversion components and signal conditioning conversion circuits (Lin et al., 2020; Stewart et al., 2022). Because the output signal of a sensor is usually weak, the signal output by the sensor usually needs to undergo signal conditioning and conversion, amplification, calculation and modulation before it can be displayed and participate in control. In some cases, sensors also require an additional auxiliary power supply to provide conversion energy. Sensitive components refer to the parts of the sensor that can directly sense or respond to the measurement. The conversion element refers to the part that can convert the electrical signal in the sensor being sensed or responded to into a suitable part for transmission or measurement. The design of a flexible wearable sensor structure includes material stacking structure, electrode layout and other aspects (Jin et al., 2018; Fan et al., 2021). This article uses copper tape to fix the conductive fiber film on both ends of the electrode and applies the obtained conjugated material compound between the conductive fiber film of the sensor and the copper tape. The purpose is to reduce the contact resistance of the two materials, optimize sensor performance and improve the sensitivity and hydrophobicity of the sensor.

#### 2.2.2 Sensor fabrication and assembly

This article uses flexible polymer materials as the matrix material and conjugated materials with excellent electrical properties as conductive fillers to prepare flexible wearable sensors with high strain and sensitivity through different methods (Zhao et al., 2022). In this paper, TPU (Thermoplastic polyurethanes) fiber membrane composed of dimensional network structure is formed inside by electrospinning. This structure can endow the TPU matrix with a large specific surface area and high tensile strength. Conjugated materials with ultra-thin structure and excellent electrochemical performance are used as conductive fillers, which are wrapped on the surface of TPU fiber by ultrasonic dispersion method. Conjugated material nanosheets are evenly distributed on the TPU fiber and overlap each other, forming a conductive channel of conjugated materials. A complete three-dimensional conduction network is formed between each conductive channel using a TPU three-dimensional network as the skeleton. The response mechanism of strain sensors based on functional polymer conjugated materials includes changes in distance between conductive fillers and changes in material geometry. Functional polymer conjugated materials have multi-level conductive network structure, wide strain range, high sensitivity and other advantages, which can comprehensively monitor human motion. For example, walking, running, jumping, arm bending, facial expression changes, pulse pulsation, voice production, etc. It provides new ideas for the development of flexible wearable sensors.

Copper tape is used as a conductive material in flexible sensors to transmit the sensor’s sensing signal to the readout circuit. The strain tolerance of copper strip refers to the range of change in its resistance value when subjected to a certain strain. This strain tolerance is an important parameter of copper strip, as it directly affects the sensitivity and data accuracy of the sensor. When the copper strip is subjected to strain (such as bending, stretching, etc.), its resistance value will change. This change is due to the change in the geometric shape and size of the copper strip under strain, resulting in a change in resistance. The smaller the strain tolerance of the copper strip, the greater the change in its resistance value when subjected to a certain strain. For flexible sensors, the strain tolerance of copper strips is an important performance indicator. If the strain tolerance of the copper strip is too large, it will cause the resistance value of the sensor to not change significantly when subjected to strain, thereby affecting the sensitivity and data accuracy of the sensor. On the contrary, if the strain tolerance of the copper strip is too small, it will cause a significant change in the resistance value of the sensor when subjected to small strains, which will also affect the data accuracy of the sensor. When designing sensors, it is also necessary to consider the impact of the strain tolerance of copper strips on sensor performance to ensure the accuracy and stability of the sensor’s readout.

#### 2.2.3 Sensor performance testing

After designing a flexible wearable sensor using functional polymer conjugated materials, the performance of the designed sensor should be tested. This article tests the tensile sensing performance of a sensor wrapped in conjugated materials to evaluate its stability, repeatability and sensitivity in small-scale tensile applications.

**FIGURE 3:**
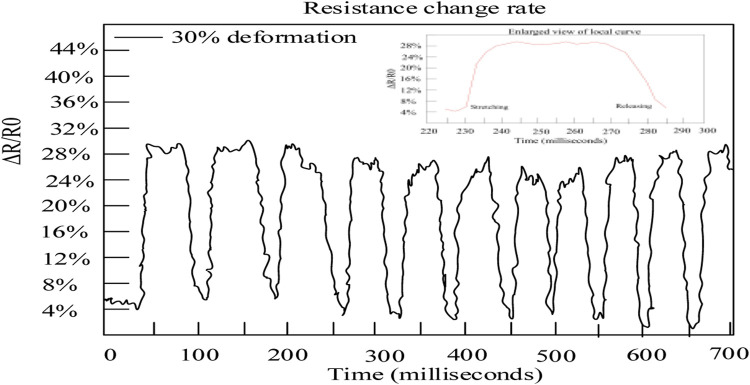
Performance test results.

Within a certain period of time, a constant current is applied to the fibers at both ends of the sensor to make the deformation of the fiber 30% and then release it evenly and slowly. Meanwhile, the voltage on both sides of the fiber is measured when repeatedly pulling and loosening at a frequency of 30 Hz and the resistance value on the fiber is calculated in combination with Ohm’s law. The results are shown in Figure 3.

The horizontal axis represents time and the vertical axis represents the relative rate of change in resistance.

According to Figure 3, the relative resistance change rate was always controlled within 32%, indicating that sensors coated with functional polymer conjugated materials had a more sensitive response to slight stretching. Moreover, under certain external forces, the amplitudes of the peaks and valleys of the functional polymer conjugated materials were basically consistent, indicating good repeatability and stability of the sensor. Concluded from the small graph in Figure 3, as the stretching amplitude increased, the resistance change rate of the sensor showed a monotonic upward trend and the overall curve was relatively smooth. Then, during the slow release process, the resistance change rate of the sensor showed a monotonic downward trend, indicating that the sensor of functional polymer conjugated materials had good linearity for small range stretching sensing. Besides, the curve corresponding to the stretching part and the curve corresponding to the release part had good symmetry. This indicates that the sensor had a relatively consistent response to its own degree of deformation, regardless of whether it was in a stretching or contracting state.

### 2.3 Design and implementation of motion monitoring system

The motion monitoring system composed of flexible wearable sensors in this article is a wireless motion monitoring system with high reliability and real-time performance. Due to the fact that people’s normal lives cannot do without exercise, a fast data collection device must be used to achieve a better exercise experience, which poses greater challenges to the motion monitoring system. Therefore, the structure of a motion monitoring system should be designed based on flexible wearable sensors to ameliorate system performance and better satisfy current demands. The system design mainly includes the connection and communication between sensors and data acquisition devices.

Flexible wearable sensors can exhibit excellent sensing characteristics when in contact with the human body (Su et al., 2022). However, in actual sports activity monitoring, the data obtained from sensor monitoring may have some deviations and are unstable. This is because during human movement, the contact surface between joints and sensors has a certain deviation and most sensors find it difficult to respond to these subtle differences. To enable flexible wearable sensors to accurately obtain human motion joints and posture changes, it is necessary to collect raw response data and perform certain processing to ameliorate the monitoring effect of the system as much as possible.

To effectively express the changes in joints and posture during human movement, the collected data should be expressed in an intuitive and efficient manner. Assuming that in sample collection, each sample can simultaneously obtain raw perceptual information from 10 perceptual units. If $U_{\text{org}\left[m\right]}$ can be used to represent the original response value of a joint, the original data obtained from each sampling can be represented as a 10 dimensional vector, whose formula is:

$$U_{\text{org}}=\left(U_{\text{org}\left[0\right]},U_{\text{org}\left[1\right]},\cdots ,U_{\text{org}\left[9\right]}\right)$$ (1)

Owing to different human body movements and varying degrees of joint bending, the response of sensors may also vary. Additionally, the sensitivity of the sensors prepared varies due to the degree of encapsulation of functional polymer conjugated materials. This results in different sensitivity of the obtained sensors to human motion posture and joint bending, leading to different response ranges of the sensors. To solve this problem, the original data must be standardized. Therefore, this article uses two maximum vectors to normalize the truncated filtered data, converting the response values of each sensor to the same dimension.

By using the above processing, the original perception data under different motion postures and joints can be mapped to the range of [0,1]. At this point, the sampled data for each frame can be represented by the formula:

$$U_{\text{nor}}=\left(u_{\text{nor}\left[0\right]},u_{\text{nor}\left[1\right]},\cdots ,u_{\text{nor}\left[9\right]}\right)$$ (2)

When collecting data on human motion posture and joints, the measurement results may have some noise due to small changes in the human body and jitter of fiber optic sensors. To filter out these noises and better express the characteristics of human motion in the data, the exponential weighted average method is chosen to filter the data (Noor-ul-Amin et al., 2020). The expression formulas for its filter are:

$$u_{1}=u_{1}^{\text{org}}$$ (3)

$$u_{r}=\gamma u_{r-1}+\left(1-\gamma \right)u_{r}^{\text{org}}$$ (4)

Among them: $u_{r}^{\text{org}}$ -data before filtering at time r; $u_{r}$ -filtered data at time r;

γ -coefficient within the range of (0,1).

The data is filtered by the above method, so that the response value at the current time point is not only related to the original data obtained by sampling, but also includes the response value obtained in a previous period of time. By assigning different weights to the data through exponential weighting and then summing and averaging them, the actual response value after filtering at the current time can be obtained. This method can not only eliminate the fluctuation of data caused by partial noise, but also alleviate the excessive response caused by external environmental factors (such as sudden large stretching/releasing). The formula can be expressed as:

$$Z=\left(\left(G_{1},j_{1}\right),\left(G_{2},j_{2}\right),\cdots ,\left(G_{I},j_{I}\right)\right)$$ (5)

Among them: I -number of samples;

$G_{1}、G_{2}、G_{I}$ -certain attitude data; $j_{1}、j_{2}、j_{I}$ -certain types of pose label to be recognized.

**FIGURE 4:**
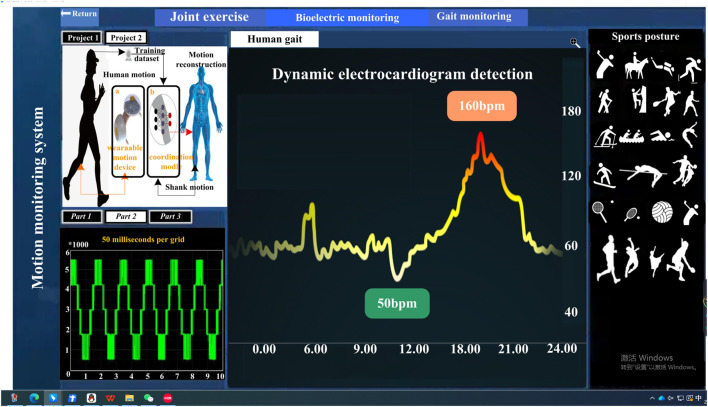
Test interface diagram of motion monitoring system.

This article takes the fiber sensor of the prepared functional polymer conjugated material as the core sensing unit, supplemented by devices such as microprocessors and analog-to-digital converters, to develop a motion monitoring system that can monitor human motion in real-time. Among them, the adaptive program formed by truncation filtering and data normalization can effectively solve measurement errors caused by differences in human posture and joint bending amplitude and also effectively diminish the negative effects caused by factors such as initial resistance and sensitivity differences of sensors. The testing interface of the motion monitoring system is shown in Figure 4.

Considering that the motion monitoring system in this article needs to meet wearability requirements, the selected hardware has a smaller volume and lighter weight. Especially for the selection of batteries, it is extremely important. The selected battery in this article has a height of 59.0 mm and a diameter of 32.3 mm. A protective board circuit is installed in the battery. When the battery experiences overcharging, discharging or short circuit, the protective circuit can isolate the battery from the circuit. The volume and thickness of the entire battery are very small. The purpose of doing this is to reduce the thickness of the final product and enable it to directly attach to the clothing. The system uses a flexible sensor made of functional polymer conjugated materials, which can monitor the real-time state of human motion and provide a basis for further analysis of human motion status.

## 3 Experimental verification and evaluation of flexible wearable sensor motion monitoring

After designing a flexible wearable sensor motion monitoring system based on new functional polymer conjugated materials, the performance of the flexible wearable sensor in motion monitoring should be evaluated to verify the performance of the system, such as accuracy, stability and real-time performance, thereby offer more basis to the practical application of new functional polymer conjugated materials in flexible wearable sensor motion monitoring.

### 3.1 Experimental design and data collection

To better validate the performance of the sports monitoring system, this article selects sophomore students from a local university’s physical education college as the research object. The flexible wearable sensor is used to collect the output data of students’ daily activities and movements, forming a sports data set. There are a total of 5000 data in this data set and 500 data for running, high jump and long jump training.

### 3.2 System performance evaluation

#### 3.2.1 Accuracy evaluation

**FIGURE 5:**
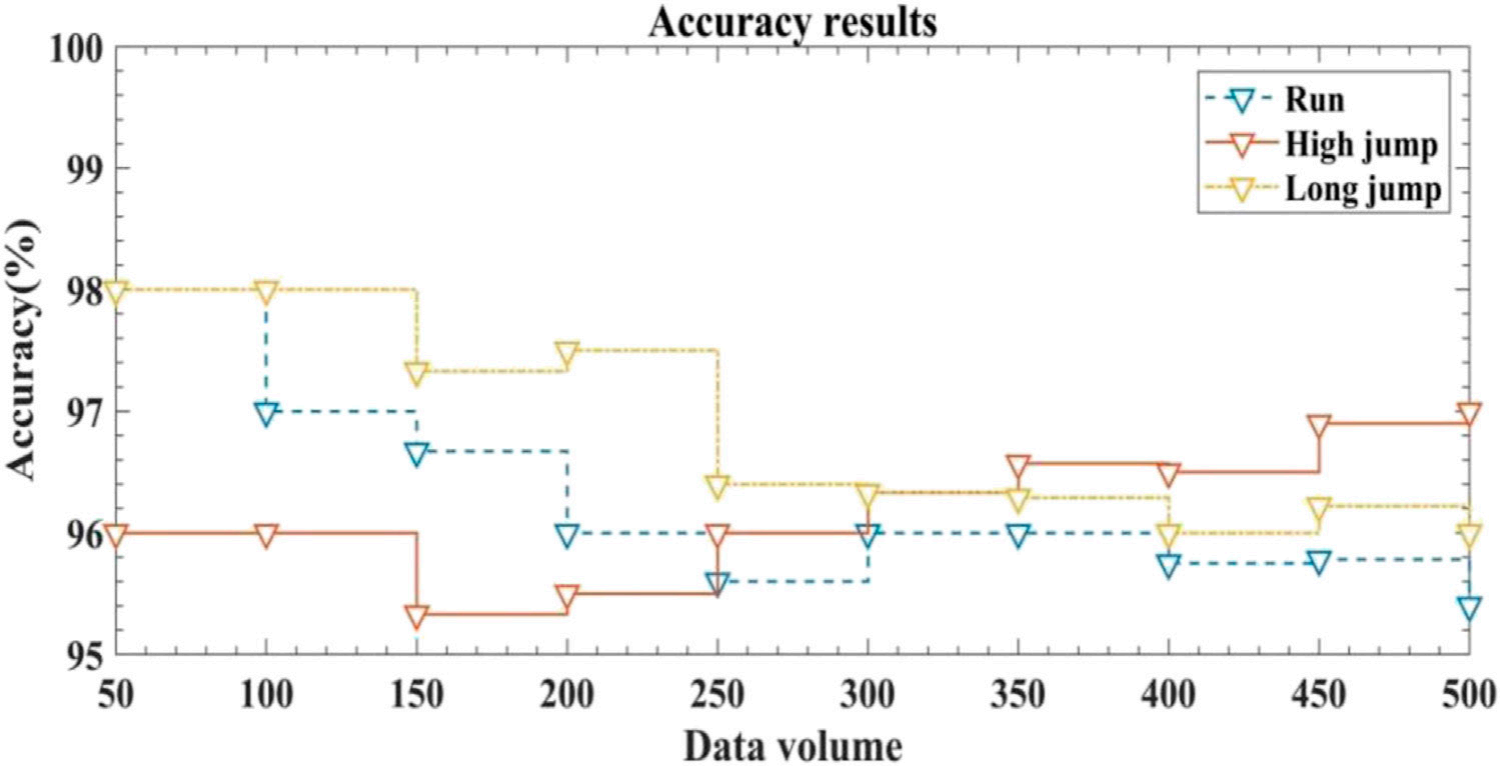
Accuracy of exercise monitoring for running, high jump and long jump training under different output data conditions.

Accuracy is the most important criterion for measuring the performance of motion monitoring systems. The higher the system accuracy, the better the system performance, which is more conducive to promoting scientific training and analysis. This article tested the accuracy of the system in monitoring running, high jump and long jump training under different output data conditions. The results are depicted in Figure 5.

The horizontal axis represents the amount of output data and the vertical axis represents the accuracy of system monitoring when outputting different amounts of data.

According to Figure 5, when the output data was 50, the accuracy of the system was 98% for running training monitoring, 96% for high jump training monitoring and 98% for long jump training monitoring. When the output data was 250, the accuracy of the system was 95.6% for running training monitoring, 96% for high jump training monitoring and 96.4% for long jump training monitoring. When the output data was 500, the system had an accuracy of 95.4% for running training monitoring, 97% for high jump training monitoring and 96% for long jump training monitoring. Overall, the accuracy of the system for motion monitoring was generally above 95%, with a high level of accuracy and reliability. This indicates that the system had good performance, could accurately monitor the state of motion training and conduct scientific analysis of the training situation, which helped to develop more reasonable motion training plans and improve the efficiency of motion training.

In addition, the accuracy of motion monitoring using the method proposed in this paper was compared with that of references 4, 5, and 7 when the output data was 500. The results are shown in Table 4.

**TABLE 4:** Comparison of accuracy of different methods at a data volume of 500.

Types of sports	This paper (%)	Literature 4 (%)	Literature 5 (%)	Literature 7 (%)
Running	95.4	91.3	79.6	80.8
High jump	97	89.5	75.4	81.3
Long jump	96	90.2	74.3	82.6

From Table 4, it can be seen that when the output data volume is 500, the monitoring accuracy of the method in this paper is the highest, followed by the method in Reference 4, followed by the method in Reference 7, and the accuracy of the method in Reference 5 is the lowest. From it, it can be seen that the method proposed in this article has the highest monitoring accuracy and can better achieve real-time monitoring of motion.

#### 3.2.2 Stability evaluation

**FIGURE 6:**
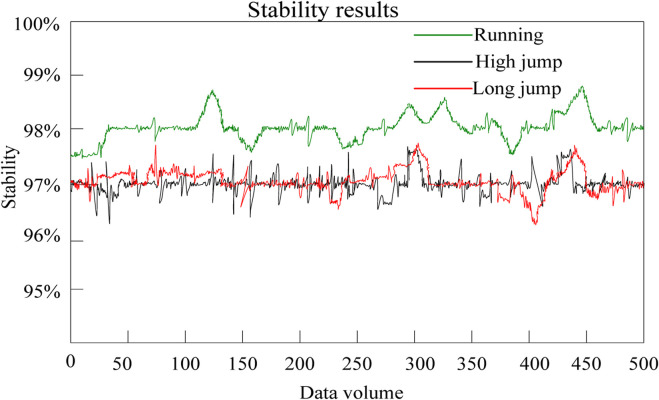
Stability test results of motion monitoring system.

In a system, stability is the key to ensuring high-quality, efficient and high-precision output. Stability is also an significant indicator for measuring the performance of motion monitoring systems. The better the stability of a flexible wearable sensor motion monitoring system, the more conducive it is to monitoring the training status and situation. This article tested the stability of the motion monitoring system. The results are depicted in Figure 6.

The horizontal axis represents the output data volume and the vertical axis represents the system stability when outputting data volume.

Seen from Figure 6, the stability of the system for running monitoring was basically controlled at around 98%, with the highest stability value being close to 99% and the lowest value being around 97.5%. Overall, the system had high stability and good stability for running monitoring without significant fluctuations. Additionally, the system had high stability in monitoring high jump and long jump movements, with changes of around 97% and maintained good stability without significant fluctuations. This indicates that the overall stability of the motion monitoring system in this article was good and it could better monitor the exercise training situation and training status. The reason is that the motion monitoring system in this article optimized the performance of flexible wearable sensors by introducing new functional polymer conjugated materials. The sensitivity and stability of the sensors increased, which improved the stability of the system and enabled more stable motion monitoring.

In order to better highlight the stability of the method proposed in this paper for motion monitoring, the stability of the motion monitoring system was also tested when the output data was 500. The results are shown in Table 4, which are consistent with the methods proposed in references 4, 5, and 7.

From Table 5, it can be seen that when the output data amount is 500, the stability of the motion monitoring system in this paper is the highest, followed by the method in Reference 3, followed by the method in Reference 7, and the stability of the motion monitoring system under the method in Reference 4 is the lowest. Compared to the motion monitoring system constructed by other methods, the motion monitoring system in this article has the best stability and can ensure comprehensive monitoring of the motion situation.

**TABLE 5:** Stability comparison of different methods at a data volume of 500.

Types of sports	This paper (%)	Literature 4 (%)	Literature 5 (%)	Literature 7 (%)
Running	98.02	80.12	85.32	82.13
High jump	97.15	79.05	84.41	82.39
Long jump	97.03	80.23	85.09	82.28

#### 3.2.3 Real time evaluation

**FIGURE 7:**
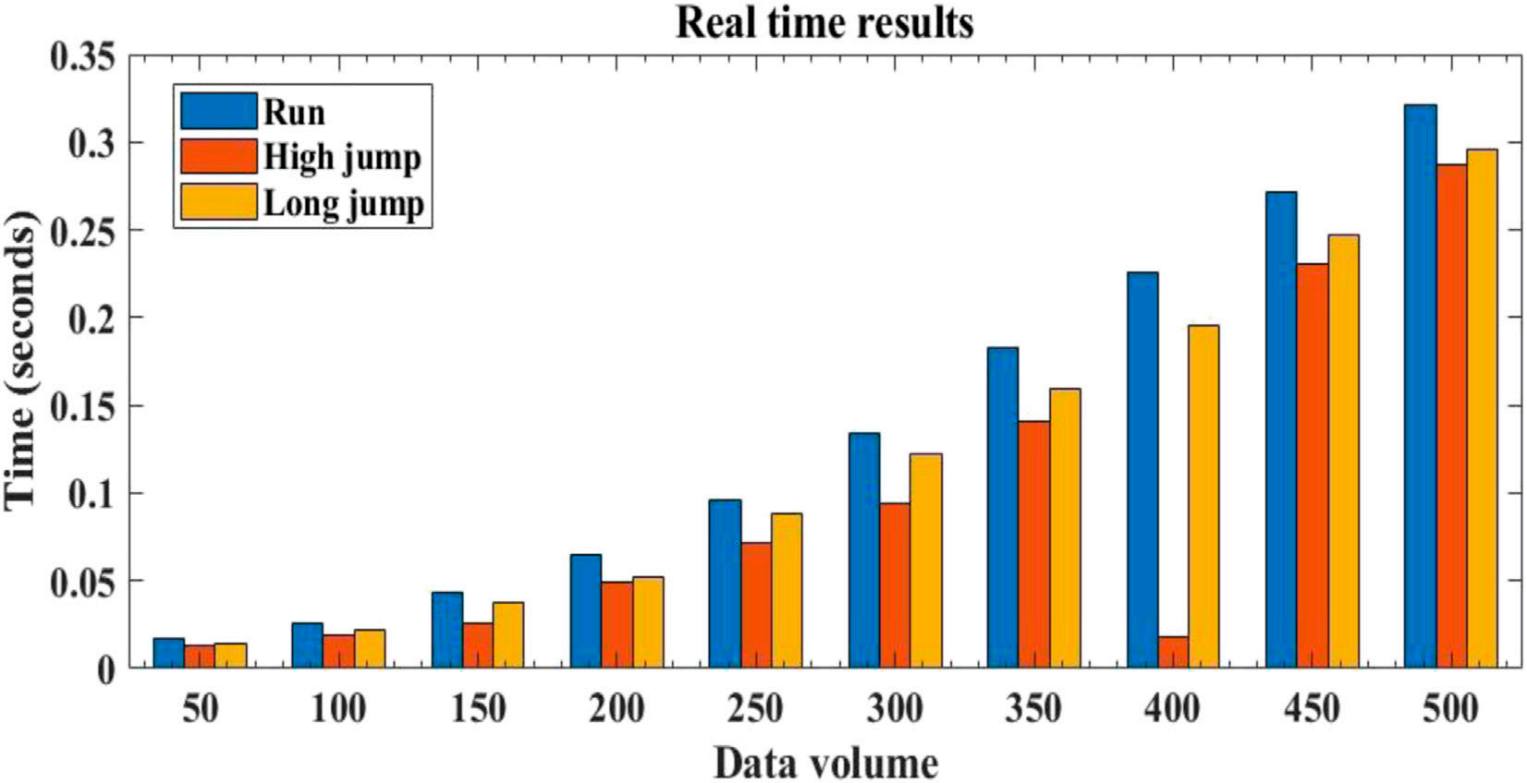
Real time test results of motion monitoring system.

Besides the above performance, the real-time performance of the motion monitoring system is also very important. A motion monitoring system with better real-time performance can constantly monitor its motion status, detect abnormal data in a timely manner and offer early warnings, which helps to achieve scientific training and increase exercise efficiency. The real-time performance of the motion monitoring system is shown in Figure 7.

The horizontal axis represents the amount of output data and the vertical axis represents the time required for system feedback when outputting different amounts of data.

Known from Figure 7, when the output data volume was 50, the system required 0.017 s for monitoring feedback on running, 0.013 s for monitoring feedback on high jump and 0.014 s for monitoring feedback on long jump. When the output data volume was 250, the system required 0.096 s for monitoring feedback on running, 0.072 s for monitoring feedback on high jump and 0.088 s for monitoring feedback on long jump. When the output data volume was 500, the system required 0.321 s for running monitoring feedback, 0.287 s for high jump monitoring feedback and 0.296 s for long jump monitoring feedback. It can be seen that as the output data volume continued to increase, the time required for system feedback also gradually increased. However, overall, the feedback time required by the system was relatively short, which meant that the real-time performance of the system in this article was good. The system can provide timely feedback on the monitored sports data, which helps athletes better identify and process abnormal data during sports training and develop more scientific sports training plans based on sports data to improve training effectiveness. The reason for this is that the sensors in the system are optimized using new functional polymer conjugated materials, which improves the sensitivity of the sensors. Meanwhile, after data collection, the system filters and processes the data to better and more accurately extract motion information and provides timely feedback on motion training information.

### 3.3 Discussion

Motion monitoring is a complex and multidimensional analytical process. Therefore, the training monitoring of athletes requires a large sports monitoring system as support, and sports monitoring is a process of quantitative analysis of physiological indicators. In this process, it is necessary for various types of sensors to work together and perform their respective functions. The performance improvement of each type of sensor can lead to a leapfrog improvement in the performance of the entire motion monitoring system.

The flexible wearable sensor of the novel functional polymer conjugated material in this article can analyze the heart and muscles during exercise by real-time monitoring of electrophysiological signals, thereby providing support for exercise rehabilitation and motion analysis. With the integration of various disciplines, the performance and functionality of sensors have greatly improved. Sensors based on electrophysiological signals can obtain more accurate electrophysiological signals and apply them to various types of motion analysis. To obtain more accurate electrophysiological signals, the most important thing is the preparation of sensor electrodes. The rationality of electrode structure design has a significant impact on the signal-to-noise ratio of the obtained signal. It is best for electrodes to have a good combination in terms of biocompatibility, softness, adhesion, conductivity, and breathability. This article is based on a flexible wearable sensor made of novel functional polymer conjugated materials, which can accurately monitor specific physiological signals. At the same time, it relies on an adaptive program formed by truncation filtering and data normalization to achieve data processing and denoising. With the development of intelligent manufacturing, the diversity and integration of sensors can be greatly improved. Integrating different sensors together can effectively solve the problem of single factor detection of sensors, thus providing more possibilities for multivariate motion monitoring.

## 4 Conclusion

Because of the continuous improvement of chemical material preparation methods, the application of conjugated materials is also becoming increasingly widespread. However, in the field of sensors, the application of conjugated materials still needs further exploration. The research direction of this article was the study of flexible wearable sensors for motion monitoring based on novel functional polymer conjugated materials. Firstly, the relevant background of its research was introduced, followed by a review of the strengths and weaknesses of previous scholars. Afterwards, this article provided a detailed analysis of the preparation and performance of novel functional polymer conjugated materials and then explored the preparation methods and performance of sensors. Based on this, combining sensors based on novel functional polymer conjugated materials, this paper designed the latest flexible wearable sensor motion monitoring system and tested its performance. The research results showed that the motion monitoring system designed in this article had good accuracy, stability and real-time performance. It could accurately and stably monitor the exercise training situation and provide timely feedback, thereby improving the effectiveness of exercise training and ensuring the smooth progress of sports. It had good practical application value. However, there are also shortcomings in the research. Due to practical limitations, the data set composed in this article is relatively small, which may have a certain influence on the final research results when selecting small data samples during system testing. In addition, the sensors prepared in this article still have significant room for improvement. In subsequent research, better and more complete conductive networks can be constructed to enhance their strain range, Meanwhile, the system is difficult to monitor small signals in the human body. In the future, more sensitive conductive networks such as crack structures and fold structures can be constructed to achieve this.

## Data Availability

The original contributions presented in the study are included in the article/Supplementary material, further inquiries can be directed to the corresponding authors.
